# PERSONALITY AND SELF-REPORTED AND ACTIGRAPHY-MEASURED SLEEP HEALTH IN ADULTHOOD

**DOI:** 10.1093/geroni/igz038.2864

**Published:** 2019-11-08

**Authors:** Nasreen A Sadeq, Nasreen A Sadeq, Soomi Lee, Alyssa Gamaldo, David M Almeida

**Affiliations:** 1 University of South Florida, Tampa, Florida, United States; 2 The Pennsylvania State University, University Park, Pennsylvania, United States

## Abstract

Personality may be associated with sleep health, however, the majority of existing studies rely on self-reported measures of sleep (often focusing on sleep duration). The purpose of this study is to examine the associations between Big Five personality traits and self-reported and actigraphy measured sleep. This study included 3928 participants and a subsample of 441 participants from the Midlife in the United States study. Linear regressions were used to analyze the relationships between personality traits and sleep. Neuroticism was associated with more frequent actigraphy-measured waking after sleep onset, and several self-reported measures of sleep quality, including shorter sleep duration, longer sleep latency, and a greater number of insomnia symptoms. Agreeableness was associated with shorter actigraphy-measured sleep duration and more self-reported insomnia symptoms. Our findings support an association between Neuroticism and poor sleep, and suggest that Agreeableness may be associated with worse sleep health.

